# Letter from Cincinnati, Ohio

**Published:** 1871-11

**Authors:** T. H. Kearney


### LETTER FROM CINCINNATI, OHIO.

BY T. H. KEARNEY, M. D., OCT. 13TH, 1871.

*Editors of The Georgia Medical Companion:*

GENTLEMEN—Though so many hundred miles away, I hope I may be permitted to express the interest I feel in my professional brethren in Georgia, as well as here, in Ohio. For citizenship in the Commonwealth of medicine, is not limited by geographical lines, nor a fraternal interest and sympathy lessened by distance. And as you, gentlemen, have paid me the compliment of associating my name with those of your correspondents in various localities, I must hasten to acknowledge that honor. Be assured then, of my full appreciation of that honor, and of my best wishes for the success of THE COMPANION.

Perhaps it may not be without interest to your readers to hear something about the public medical institutions of Cincinnati. In this city, of some 220,000 inhabitants, there are three medical colleges, viz: The "Medical College of Ohio," the "Miami Medical College," and the "Cincinnati College of Medicine and Surgery." The first named is the oldest, having celebrated its semicentenial last Spring. For a long time it enjoyed a monopoly of medical teaching here, and its faculty has been ornamented, from time to time, by some of the brightest men of the profession, in the country. But of late years, it has had to divide the patronage with its vigorous and wide-awake rivals. The Miami College, although many years the junior of the Ohio, is fully its equal in prosperity and reputation at the present time. Both these schools commenced the present session with uncommonly large classes.

Competition in medical teaching leads to some good results, by the activity and zeal it is so apt to induce in the individual teachers. But, on the other hand, it must be admitted, it tends to disadvantages also, by lowering the standard of qualifications requisite of the student. In such a country as curs, where there is no legal control over such matters, such competition will almost inevitably tend to the lowering of that standard. For medical corporations are like individuals,—one feels justified in doing what a rival does, on the *self-preservation* principle. Thus it is that, from time to time, the fees are lowered by some one, *compelling* the others to follow suit. At this time the fees in Cincinnati are little more than one-third what they were twelve or fourteen years ago. The gentlemen who compose the faculties of our colleges are certainly entitled to something like reasonable compensation for their services ; for, very generally, they bring to the discharge of their professional duties first-class abilities, zeal and industry. In contrast with the beggarly forty dollars charged for a course of lectures in the medical schools, it must be stated that the Dental College, in this same city, charges one hundred dollars. It is to be hoped no one will argue from these figures that dentistry, in any sense, stands to the profession of medicine in the ratio of one hundred to forty ! Even an irregular establishment here, charges fifty per cent. more than the regular schools do. All this is wrong. As a matter of business policy, it is a mistake; for our colleges here, may as well have fair prices for their tuition as such ridiculously inadequate figures. Indeed, such a glaring disproportion between our charges here and those in the Eastern cities, must often be taken for an acknowledgment of corresponding inferiority. Any such inference I protest against; for, at the present day, the advantages offered by Cincinnati, as a school of medicine, are hardly inferior to those of any city in the country. In the particular of clinical teaching especially, great advances have been made within the last few years. And the different important specialties are now thoroughly taught here, instead of being superficially treated in connexion with some general subject, as was formerly the case. Certain of these subjects are taught in the Spring courses, (now held regularly in all of the schools,) the regular Winter course not affording the necessary time. Another highly important aid to thorough teaching, which is in vogue, is the system of "quiz classes," or private examinations, by which the substance of the lecture is repeated, obscure points exemplified, etc.; and thus the subjects of study are more thoroughly impressed on the minds of students.

The principal field for clinical study, is the new Cincinnati Hospital, completed a couple a years ago, at a cost of over three quarters of a million of dollars. This magnificent structure, consisting of six separate pavilions of three stories, with the central block or administrative buildings, and the kitchens, laundry, etc., is arranged in the form of a hollow square. The main building fronts on twelfth street; the kitchen and other offices, constituting the rear, extend on Ann street. The pavilions on the west side, look out on Central avenue, while those on the east, front on the canal. The establishment, thus occupying an entire block to itself, is very favorably situated as regards air and light. The plan upon which it is constructed embodies the most recent improvements in ventilation and heating. The former being accomplished by a system of flues, registers and air ducts, which communicate with the smokestack in the rear, through which the exhausted or foul air is discharged. The heating is by means of coils of steam pipe, supplied from boilers in the rear. The capacity of the building is between five and six hundred beds. And the lecture and operation theatre will seat six hundred students. Two lectures are delivered in the hospital each week day. Besides medicine and surgery, the subjects of gynecology, diseases of children, ophthalmology and pathology are taught in these lectures. And the students, in small numbers at a time, are allowed to observe cases of labor at the bedside, under the instruction of one of the obstetricians. In like manner, the pathologists afford the class apportunities of seeing *post mortem* examinations made. Thus, it will be seen the clinical advantages here are very ample—indeed more so than in any hospital in the country. But, in addition, each of the schools has a dispensary attached to it, which affords for study a class of cases differing from those requiring hospital treatment, and so giving a wider variety to the clinical material. In addition to the city hospital first mentioned, there are, also, the "Hospital of the Good Samaritan," and "St. Mary's" Hospital, both under the control of the Sisters of Charity, and a small one belonging to the Hebrews. At the Good Samaritan, clinical lectures are also given twice a week.

One thing Cincinnatians are becoming very proud of, is the Public Library, which is fast attaining larger proportions, and the magnificent new building for which is approaching completion. Here is a separate medical department, which, of course, is a great advantage to the profession. And it promises to become more and more so, under a liberal law lately enacted, which appropriates the proceeds of the sale of the hospital tickets to its maintainance and increase. On the tables of its reading room are now to be found nearly all the medical periodicals in the English language, with the chief of the French and German journals. We hope to see the GEORGIA MEDICAL COMPANION among them in the future. At some future time I hope to be able to contribute to your journal something of more professional interest than this gossipy and unintentionally long letter.

